# A Case of Diffuse Pulmonary Ossification Complicated by Obstructive Pneumonia

**DOI:** 10.5761/atcs.cr.26-00096

**Published:** 2026-10-24

**Authors:** Kazuki Sugata, Yoshitaka Tanaka, Hiroki Katsushima, Yoshio Tsunezuka

**Affiliations:** 1 Department of General Thoracic Surgery, Saneikai Tsukazaki Hospital, Himeji, Hyogo, Japan; 2 Department of General Internal Medicine, Saneikai Hirohata Hospital, Himeji, Hyogo, Japan; 3 Department of Pathology, Saneikai Tsukazaki Hospital, Himeji, Hyogo, Japan

**Keywords:** diffuse pulmonary ossification, obstructive pneumonia, bronchial obstruction, progressive pulmonary ossification

## Abstract

Diffuse pulmonary ossification (DPO) is a rare and usually asymptomatic condition characterized by ectopic bone formation in the lungs. A 45-year-old man presented with a low-grade fever, cough, and fatigue. Chest computed tomography revealed a calcified lesion near the left B9 bronchus with bronchial narrowing and distal consolidation, as well as multiple small calcified nodules in the upper and lower lobes of both lungs. Bronchoscopy revealed complete obstruction of the left B9 bronchus. Transbronchial biopsy revealed cartilaginous tissue without malignancy. Despite antibiotic therapy, the symptoms persisted, and malignancy could not be excluded. Subsequently, thoracoscopic surgery was performed. Due to the strong adhesion between the lesion and the bronchus, a left basal segmentectomy was required. Histopathological examination revealed mature trabecular bone with marrow elements, consistent with pulmonary ossification. Although generally benign, pulmonary ossification can cause bronchial obstruction and refractory pneumonia and occasionally require surgical intervention.

## Introduction

Diffuse pulmonary ossification (DPO) is a rare condition characterized by ossifying lesions in the lung parenchyma.^1)^ Although the condition is classified into nodular pulmonary ossification and dendriform pulmonary ossification types based on the pattern of ossification, most cases follow an asymptomatic course and are often discovered incidentally during autopsy^2,3)^; however, there are also reports of cases requiring surgical treatment,^4–10)^ and their clinical significance remains unclear. Here, we report a case of diffuse pulmonary ossification in which the bronchus was compressed by a portion of the ossified lesion, leading to refractory obstructive pneumonia requiring surgical treatment.

## Case Report

A 45-year-old man presented with a history of low-grade fever, cough, and fatigue for several months. Chest radiography revealed an abnormal shadow in the left lung (**Fig. 1A**), and the patient was referred to our hospital for further evaluation. He had a smoking history of 20 cigarettes per day for 24 years. Laboratory findings showed a white blood cell count of 12300/μL, a C-reactive protein level of 12.5 mg/dL, and a β-D-glucan level of 7.1 pg/mL. The T-SPOT TB test result was negative. Levels of tumor markers were within normal limits (CEA 2.1 ng/mL, Pro-GRP 22.1 pg/mL, and CYFRA 1.34 ng/mL). Pulmonary function tests demonstrated a forced vital capacity (FVC) of 4.04 L (97.3% of predicted), a forced expiratory volume in 1 second (FEV_1_) of 2.98 L (83.1% of predicted), an FEV_1_/FVC ratio of 73.8%, and a diffusing capacity of the lung for carbon monoxide (DLCO) of 70.6%. Chest computed tomography revealed a calcified lesion measuring 1.2 cm in maximum diameter, adjacent to the left B9 bronchus. Narrowing of the left B9 bronchus and distal spread of infiltrates from the nodular lesion were observed, leading to a diagnosis of obstructive pneumonia (**Fig. 1B** and **1C**). Furthermore, multiple small, high-attenuation nodules were scattered throughout the upper and lower lobes of both lungs. Bronchoscopy revealed complete obstruction of the left B9 bronchus by a compressive lesion originating from outside the bronchial wall (**Fig. 1D**). Histopathological examination of the biopsy specimens revealed cartilaginous tissue without evidence of malignancy. Despite antibiotic therapy, the patient’s symptoms showed minimal improvement. Given the possibility of a malignant tumor causing the bronchial obstruction, surgical intervention was planned. Thoracoscopic left S9 segmentectomy was initially planned. Intraoperatively, firm adhesion between the ossifying lesion and the left B9 bronchus made dissection difficult; therefore, a left basal segmentectomy was performed instead. The operative time was 2 h 59 min, and the intraoperative blood loss was 20 mL. The postoperative course was uneventful, and the patient was discharged on postoperative day 4.

**Fig. 1:**
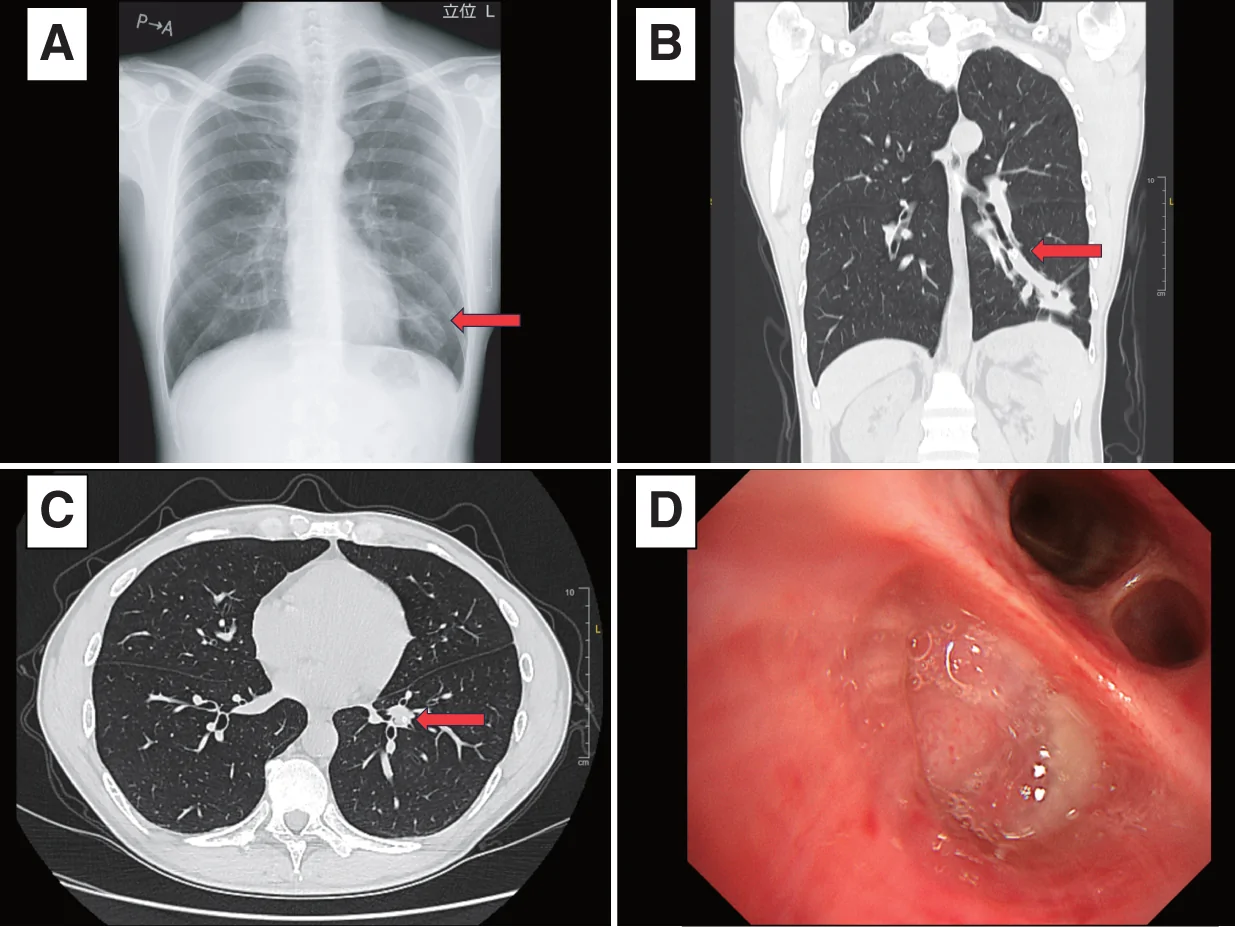
Chest imaging and bronchoscopic image. (**A**) An infiltrative shadow was observed in the left lower lung field (red arrow). (**B**) Mucoid impaction distal to the obstruction of B9 with surrounding inflammatory changes was observed. The red arrow indicates an ossified nodule. (**C**) A high-attenuation nodular lesion caused complete obstruction of the left B9 bronchus. The red arrow indicates an ossified nodule. (**D**) The left B9 bronchus was completely obstructed by an occupying lesion, as shown in the bronchoscopic image

The histopathological examination revealed pulmonary ossification. The left B9 bronchus was completely obstructed by a nodule extending from the lung parenchyma (**Fig. 2A** and **2B**). The nodules comprised the trabecular bone, cellular marrow, and fatty marrow, consistent with ossifying lesions (**Fig. 2C**). Additionally, fibrous tissue proliferation and lymphocytic infiltration were observed between the ossifying lesion and the bronchus (**Fig. 2D**).

**Fig. 2:**
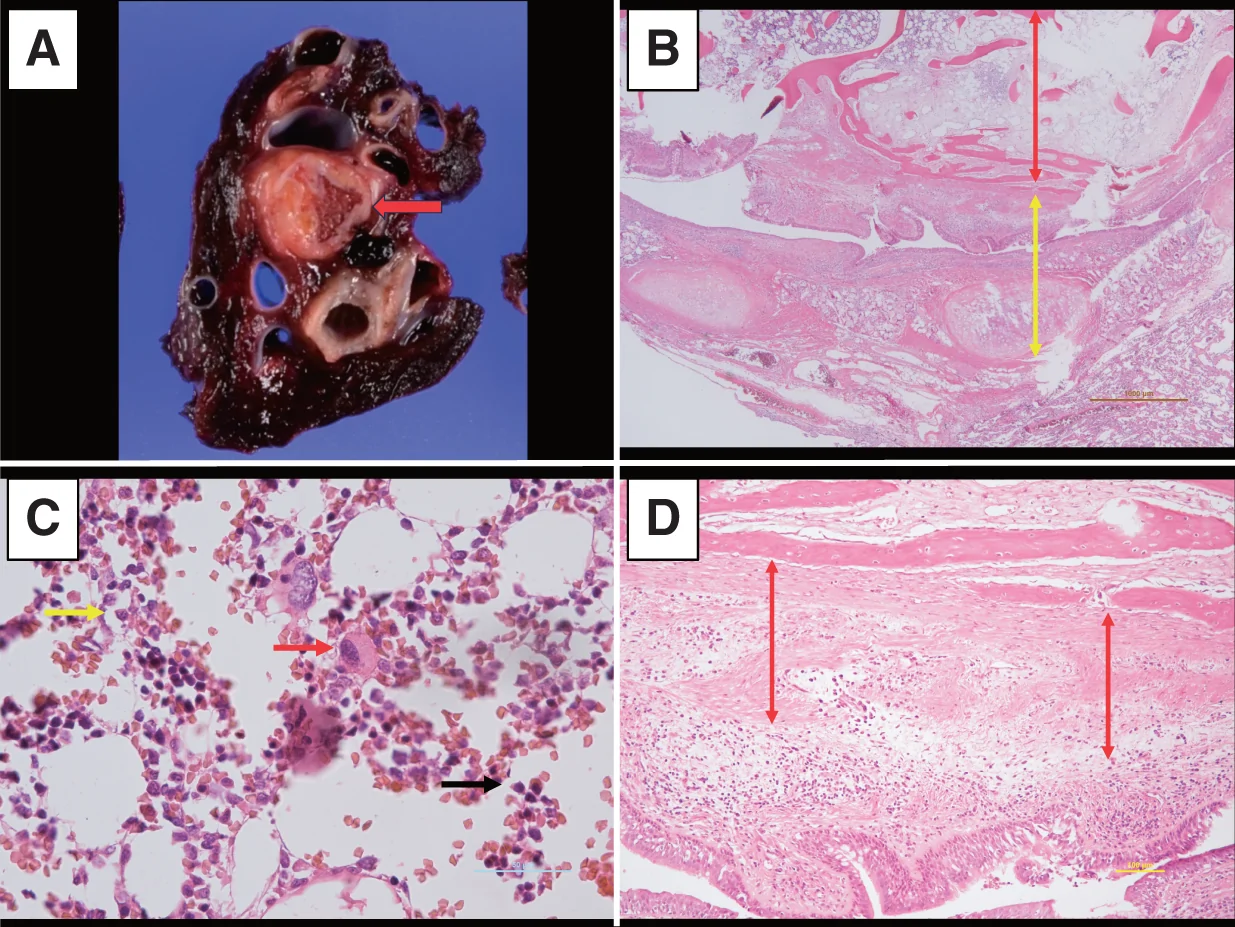
Resected lung specimen and H&E staining. (**A**) Complete obstruction of B9 due to a pulmonary ossification nodule (red arrow). (**B**) Ossification was observed within the pulmonary parenchyma. The ossified lesion (red arrow) compressed the bronchus (yellow arrow) from outside the bronchial wall. (**C**) Cellular marrow within the ossified lesion. The red, black, and yellow arrows represent megakaryocytes, erythroblasts, and myeloblasts, respectively. (**D**) Proliferation of fibrous tissue (red arrow) and lymphocytic infiltration were observed between the bronchus and the osseous lesion. H&E, hematoxylin–eosin

## Discussion

This case report highlights 2 important clinical considerations. First, pulmonary ossification can result in clinically significant complications depending on its anatomical location and extent. Second, when surgical intervention is required, anatomical lung resection may be technically challenging because of the dense adhesions between the ossified lesions and the surrounding tissues.

Although DPO is often asymptomatic and incidentally detected,^2,3)^ it may occasionally present with clinical symptoms. Surgical management of DPO has been reported in cases complicated by pneumothorax or intrapulmonary hematoma.^4–9)^ Kato and Abe described a case of pulmonary ossification associated with pneumothorax in which a subpleural ossified lesion caused pleural injury, resulting in pneumothorax.^4,5)^ Ohsumi et al. reported a case of familial DPO in which the patient underwent lung transplantation because of a progressive decline in lung function over time.^10)^ In the present case, a pulmonary ossification lesion completely obstructed the left B9 bronchus, leading to refractory obstructive pneumonia, ultimately necessitating surgical intervention.

The pathogenesis and triggers of pulmonary ossification progression remain poorly understood. The clinical course of ossifying lesions is generally considered to be slowly progressive.^11)^ Tsuchiya et al. reported gradual enlargement of pulmonary ossification lesions on computed tomography over 8 years.^12)^ In the present case, it is highly likely that the progressive enlargement of the ossifying lesion eventually led to complete obstruction of the bronchus, resulting in pneumonia, suggesting a similarly slow progression over time.

Regarding the underlying mechanisms, nodular pulmonary ossification is frequently associated with chronic cardiac conditions such as mitral stenosis and pulmonary edema. Chronic pulmonary venous congestion and recurrent alveolar hemorrhage are thought to induce the differentiation of fibroblasts into osteoblasts. In contrast, dendriform pulmonary ossification is often associated with chronic pulmonary diseases, including chronic obstructive pulmonary disease and interstitial lung disease, in which inflammation and fibrosis may promote osteoblastic activity through transforming growth factor-β (TGF-β)–mediated pathways. However, the underlying mechanisms remain hypothetical.^13–15)^ In the present case, although the patient had a history of smoking, no other underlying conditions previously reported to be associated with the pulmonary ossification were identified.

Cases of pulmonary ossification causing bronchial obstruction may pose significant challenges during anatomical lung resection and, therefore, require careful intraoperative consideration. In the present case, dense adhesions were observed between the ossified lesion, the left B9 bronchus, and the pulmonary artery (**Fig. 2D**). Consequently, dissection at the planned levels of B9 and A9 was not feasible, necessitating division of the bronchus and pulmonary artery at a more proximal level.

## Conclusion

Although pulmonary ossification is generally considered a benign condition, it may result in clinically significant complications, depending on the location and progression of the ossifying lesions. Herein, we report a clinically significant case in which an ossifying lesion compressed the bronchus, resulting in obstructive pneumonia.

## Declarations

### Data availability

The datasets used and/or analyzed in the current study are available from the corresponding author upon reasonable request.
